# *In silico* and experimental analyses predict the therapeutic value of an EZH2 inhibitor GSK343 against hepatocellular carcinoma through the induction of metallothionein genes

**DOI:** 10.18632/oncoscience.285

**Published:** 2016-01-29

**Authors:** Tsang-Pai Liu, Yi-Han Hong, Kwang-Yi Tung, Pei-Ming Yang

**Affiliations:** ^1^ The Ph.D. Program for Cancer Biology and Drug Discovery, College of Medical Science and Technology, Taipei Medical University, Taipei, Taiwan; ^2^ Department of Surgery, Mackay Memorial Hospital, Taipei, Taiwan; ^3^ Mackay Junior College of Medicine, Nursing and Management, New Taipei City, Taiwan; ^4^ Department of Medicine, Mackay Medical College, New Taipei City, Taiwan; ^5^ Liver Medical Center, Mackay Memorial Hospital, Taipei, Taiwan

**Keywords:** EZH2, metallothionein, hepatocellular carcinoma, microarray analysis, therapeutic biomarker

## Abstract

There are currently no effective molecular targeted therapies for hepatocellular carcinoma (HCC), the third leading cause of cancer-related death worldwide. Enhancer of zeste homolog 2 (EZH2), a histone H3 lysine 27 (H3K27)-specific methyltransferase, has been emerged as novel anticancer target. Our previous study has demonstrated that GSK343, an S-adenosyl-*L*-methionine (SAM)-competitive inhibitor of EZH2, induces autophagy and enhances drug sensitivity in cancer cells including HCC. In this study, an *in silico* study was performed and found that EZH2 was overexpressed in cancerous tissues of HCC patients at both gene and protein levels. Microarray analysis and *in vitro* experiments indicated that the anti-HCC activity of GSK343 was associated with the induction of metallothionein (MT) genes. In addition, the negative association of EZH2 and MT1/MT2A genes in cancer cell lines and tissues was found in public gene expression database. Taken together, our findings suggest that EZH2 inhibitors could be a good therapeutic option for HCC, and induction of MT genes was associated with the anti-HCC activity of EZH2 inhibitors.

## INTRODUCTION

Surgical resection and liver transplantation are the main curative treatments for hepatocellular carcinoma (HCC); however, only 15 to 25% of patients are suitable for these treatments [1]. In addition, HCC is relatively chemo-resistant and highly refractory to cytotoxic chemotherapy, and there is currently no reliable and effective therapy for patients with advanced or metastatic disease [1]. Therefore, HCC is still the third leading cause of cancer-related death worldwide [2]. Molecular targeted agents have been regarded as new treatment option. The multi-kinase inhibitor, sorafenib, has been approved for treating advanced HCC in 2007 [3]. However, sorafenib monotherapy seems insufficient to reach satisfactory results in HCC patients because it confers less than 3 months of actual survival gain in both Western and Asian populations [3, 4]. Therefore, it is still urgent to develop an effective therapeutic strategy for HCC.

Ehancer of zeste homolog 2 (EZH2) is a histone H3 lysine 27 (H3K27)-specific methyltransferase, mediating mono-, di- and tri-methylation at histone H3K27 (H3K27-me1/2/3) together with its interacting partners SUZ12 and EED. EZH2 is frequently overexpressed in tumors [5-7], and inhibition of EZH2 serves as potential anticancer treatments [8, 9]. Several potent EZH2 inhibitors have been developed in recent years [10]. For example, 3-Deazaneplanocin A (DZNep), a S-adenosyl- *L*-homocysteine (SAH) hydrolase inhibitor, indirectly inhibits EZH2 through the depletion of EZH2 protein and the associated H3K27-me3 [9]. One of the major class of EZH2 inhibitors belongs to the competitive inhibitors of S-adenosyl-*L*-methionine (SAM) that is a universal methyl donor for the catalytic reaction of histone methyltransferases. Several SAM-competitive inhibitors, such as EPZ005687, EI1, GSK126, and GSK343, are developed and can selectively kill lymphoma cells with EZH2-activating mutations [11–14].

Based on the *in silico* and *in vitro* analyses of this study, we found that EZH2 is overexpressed in HCC and may be an attractive molecular target for treating HCC. An EZH2 inhibitor, GSK343, acted as a potent anti-HCC agent. Microarray gene expression profiling showed the induction of metallothionein (MT) genes by GSK343, which was associated with its anticancer activity. In addition, negative association of EZH2 and MT1/MT2A expression was observed. Our study provides a novel aspect of EZH2 inhibitors for treating HCC.

## RESULTS AND DISCUSSION

### Identification of EZH2 as a therapeutic target for HCC treatment

**Figure 1:**
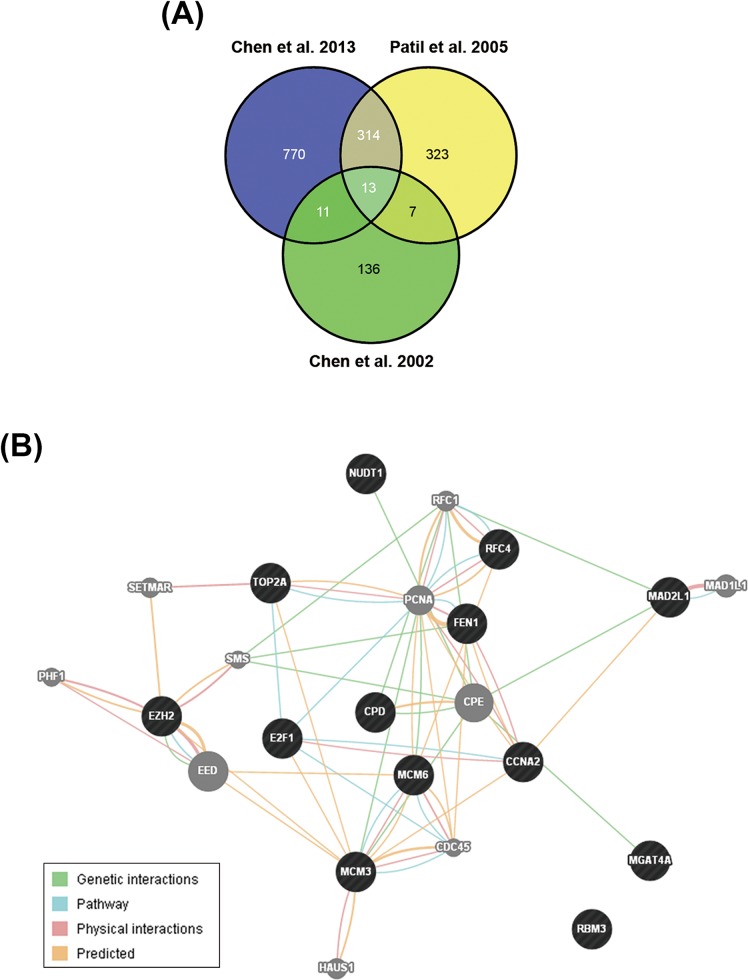
Identification of EZH2 as a potential therapeutic target for HCC treatment **A.** The Venn diagram for gene expression profiles between normal and tumor liver tissues from three published microarray datasets. The overlapped genes were shown in Table 1. **B.** Functional association of the genes in Table 1 was analyzed using the GeneMANIA (http://genemania.org/). The genes in black circles stood for the query genes. The genes in grey circles stood for the genes related to query genes.

To identify possible candidate genes essential for HCC pathogenesis, we analyzed gene expression profiles between normal and tumor liver tissues from three published microarray datasets [15-17]. The results found that 13 genes are upregulated in tumor tissues in these datasets (Figure 1A and Table 1). Their functional association was analyzed by the GeneMANIA (http://genemania.org/) [18]. Most of them are associated with cell cycle regulation (as marked by red color in Table 1), suggesting that HCC may be resulted from dysregulation of cell cycle. In addition, several genes have been reported to correlate with HCC pathogenesis, For example, deregulation of E2F1 has been implicated in the development of HCC [19]. TOP2A overexpression in HCC correlates with shorter patient survival and chemoresistance [20]. Pathway analysis showed the relationship between EZH2 and other genes in a direct or indirect manner (Figure 1B), implying EZH2 may have similar function with these genes. Indeed, EZH2 has been linked to cell cycle machinery through cyclin-dependent kinases 1/2 (CDK1/2)-dependent phosphorylation at Thr350. Blockage of Thr350 phosphorylation reduces EZH2-mediated cell proliferation and migration [21]. Therefore, we proposed that EZH2 may also participate in HCC pathogenesis. Consistently, recent studies have shown that EZH2 plays an important role in HCC tumorigenesis [22-24]. Overexpression of EZH2 is frequently detected in HCC tissues, which was correlated with the aggressiveness and poor prognosis [36-38]. Knockdown of EZH2 expression in HCC cells can reverse tumorigenicity in a nude mouse model [25], demonstrating the potential therapeutic value of EZH2 inhibition in HCC.

**Table 1:** The overlapped gene profile that is upregulated in HCC tumor tissues in three published microarray datasets

Gene Symbol	Description	Fold Change (Chen X et al.)	Fold Change (Patil MA et al.)	Fold Change (Chen YL et al.)
RBM3	RNA binding motif (RNP1, RRM) protein 3	1.569	1.563	2.246
CPD	carboxypeptidase D	1.764	1.95	1.964
CCNA2	cyclin A2	1.991	2.685	2.4
E2F1	E2F transcription factor 1	3.132	2.518	3.049
**EZH2**	enhancer of zeste homolog 2 (Drosophila)	1.924	1.952	2.565
TOP2A	topoisomerase (DNA) II alpha 170kDa	2.156	2.227	5.53
MAD2L1	MAD2 mitotic arrest deficient-like 1 (yeast)	2.474	2.712	3.381
NUDT1	nudix (nucleoside diphosphate linked moiety X)-type motif 1	2.151	2.334	2.019
FEN1	flap structure-specific endonuclease 1	2.406	2.794	2.034
RFC4	replication factor C (activator 1) 4, 37kDa	2.014	2.094	2.426
MCM3	minichromosome maintenance complex component 3	1.675	1.753	2.27
MCM6	minichromosome maintenance complex component 6	1.969	1.829	2.436
MGAT4A	Mannosyl (alpha-1,3-)-glycoprotein beta-1,4-N- acetylglucosaminyltransferase, isozyme A	1.684	1.738	2.124

EZH2 gene was highlighted in bold and cell cycle-related genes were highlighted in red color.

### Overexpression of EZH2 in HCC tissues

**Figure 2:**
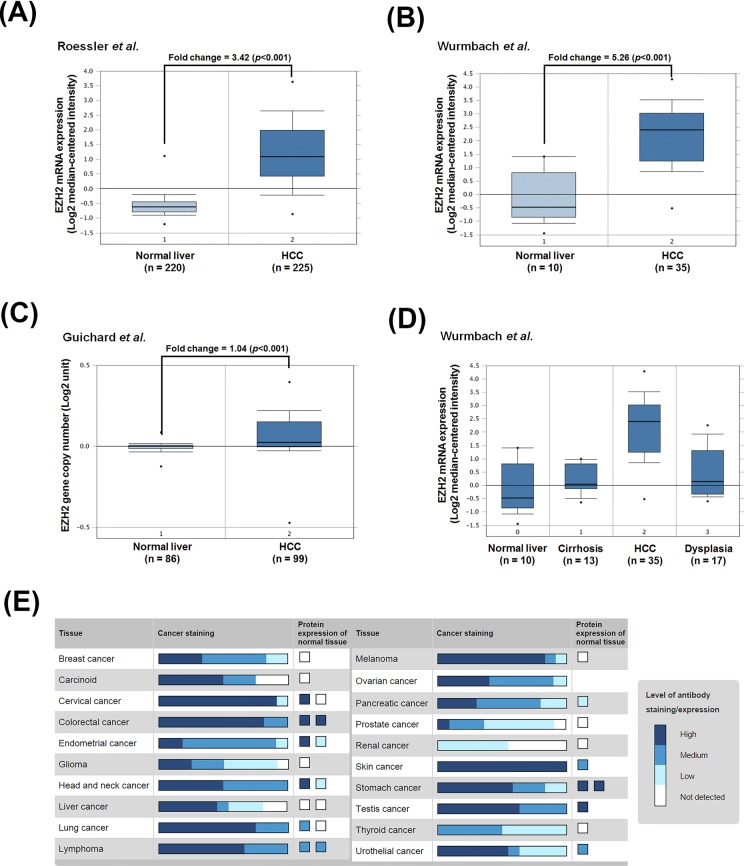
The gene and protein expressions of EZH2 in HCC patients **A**.-**C.** The copy number and mRNA expression of EZH2 in normal liver and tumor tissues. **D.** EZH2 mRNA expression in tissues of normal, cirrhosis, dysplasia, and HCC patients. Microarray datasets were obtained from the Oncomine database (http://www.oncomine.org/). **E.** EZH2 protein expression in normal liver and tumor tissues were obtained from the Human Protein Atlas (http://www.proteinatlas.org/).

To confirm the relationship of EZH2 and HCC pathogenesis, we analyzed EZH2 expression profiles using existing cDNA microarray datasets deposited in the Oncomine database (http://www.oncomine.org/) [26]. In three microarray datasets having both HCC and normal liver tissues [27-29], significantly increased EZH2 gene copy number and mRNA expression in HCC compared with normal liver tissues were found (Figures 2A-2C). In addition, Wurmbach's dataset also shows the upregulation of EZH2 mRNA in samples from tissues of normal, cirrhosis, dysplasia, to HCC in a stepwise manner (Figure 2D). To investigate whether EZH2 was also overexpressed at protein level, the immunohistochemical staining of EZH2 in normal and cancer tissues was obtained from the Human Protein Atlas (http://www.proteinatlas.org/) that is a public database with millions of immunohistochemical images in 44 different normal human tissues and 20 different cancer types, as well as 46 different human cell lines [30-34]. As shown in Figure 2E, the protein expression of EZH2 was undetectable in normal liver tissues. In liver cancer tissues, however, 6 out of 11 cases are high/medium EZH2 staining and 5 out of 11 cases are low or undetectable. These results suggest that EZH2 is overexpressed in HCC and may be an attractive molecular target for treating HCC.

### GSK343, an EZH2 inhibitor, induces metallothionein (MT) expression that is correlated with its anticancer activity

**Figure 3:**
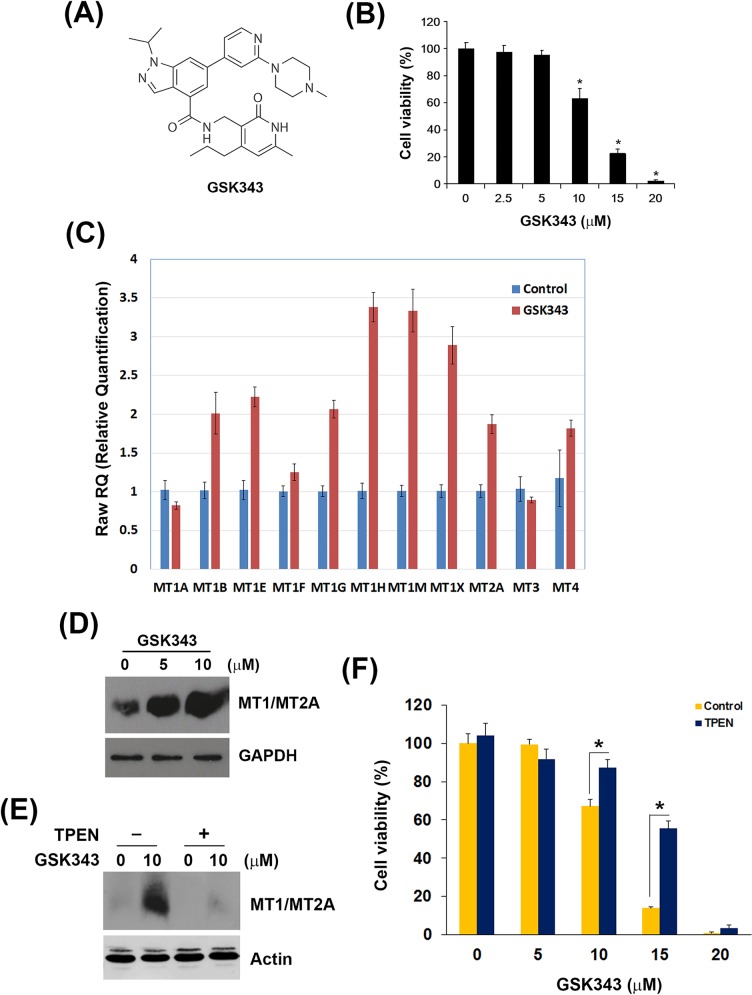
Effect of GSK343 on the cell viability and MT gene expression **A.** The chemical structure of GSK343. **B.** HepG2 cells were treated with 0-20 μM GSK343 for 72 h. Cell viability was examined by MTT assay. **C.** HepG2 cells were treated with 10 μM GSK343 for 6 h. The mRNA expression of MT1 subtypes, MT2A, MT3 and MT4 was analyzed by real-time PCR. **D.** HepG2 cells were treated with 10 μM GSK343 for 24 h. The protein expression of MT1/MT2A was analyzed by western blot analysis. **E.** HepG2 cells were treated with 10 μM GSK343 with or without 5μM TPEN for 24 h. The protein expression of MT1/MT2A was analyzed by western blot analysis. **F.** HepG2 cells were treated with 0-20 μM GSK343 with or without 5μM TPEN for 72 h. Cell viability was examined by MTT assay.

Small-molecule-based inhibition of EZH2 has recently been developed as an effective mechanism of therapeutic intervention in hematologic and solid tumors [12, 35]. Our previous study shows that an activity-based EZH2 inhibitor, GSK343 (Figure 3A), induces autophagy and enhances drug sensitivity in cancer cells including HCC. In this study, we also found that GSK343 inhibited cell viability of a human HCC cell lines, HepG2, in a dose-dependent manner (Figure 3B). To investigate the molecular mechanism involved in the anti-HCC activity of GSK343, microarray analysis was performed and found that 46 gene probes were upregulated and 123 gene probes were downregulated (Supplementary File S1). Top 10 up- and down-regulated gene probes were listed in Table 2. Interestingly, several genes (highlighted in bold) encode metallothionein 1 (MT1) subtypes and MT2 (MT2A) were induced by GSK343. MTs belong a group of cysteine-rich and low-molecular weight intracellular proteins. Due to their rich thiol content, MTs bind many trace metals such as zinc, cadmium, mercury, platinum and silver, thus protecting cells and tissues against heavy metal toxicity [36]. MT1 and MT2 (MT2A), two major isoforms of MT, are found in all types of tissues. Two other members, MT3 and MT4, are expressed in specific tissues such as brain [36]. Human MT genes are located on chromosome 16 q13 in a cluster, in which the MT2A, MT3 and MT4 proteins are encoded by a single gene, and the MT1 protein comprises many subtypes encoded by a set of MT1 genes. The known functional MT1 subtypes are MT1A, MT1B, MT1E, MT1F, MT1G, MT1H, MT1M, and MT1X, which are believed to play distinct roles, depending on their tissue-specific expression pattern [37]. The mRNA expression of all MT isoforms was examined by real-time PCR (Figure 3C). MT1 subtypes, except for MT1A and MT1F, were induced by GSK343. In addition, MT2A, but not MT3 and MT4, was also induced (Figure 3C). In addition, the induction of MT1/MT2A protein expression was also validated by western blot analysis (Figure 3D).

**Table 2:** Top 10 up- and down-regulated gene probes in HepG2 cells treated with 10 μM GSK343 for 6 h

Probe ID	Gene Symbol	Description	Fold Change
PH_hs_0028852	**MT1B**	metallothionein 1B	4.465048
PH_hs_0006434	VPS13C	vacuolar protein sorting 13 homolog C (S. cerevisiae)	3.608639
PH_hs_0006739	TMEM63C	transmembrane protein 63C	3.248965
PH_hs_0042917	**MT1M|MT1JP**	metallothionein 1Mmetallothionein 1J, pseudogene	3.151759
PH_hs_0023522	MMP25	matrix metallopeptidase 25	1.978682
PH_hs_0043618	LRTOMT	leucine rich transmembrane and 0-methyltransferase domain containing	1.926359
PH_hs_0049631	**MT1IP**|**MT1X** |**MT1E**|**MT1A** |**MT2A**|**MT1B**| |**MT1L**|**MT1H**	metallothionein 1I, pseudogenemetallothionein 1X|metallothionein 1E|metallothionein 1A|metallothionein 2A|metallothionein 1B|metallothionein 1L (gene/pseudogene)|metallothionein 1H	1.809486
PH_hs_0036496	TWIST2	twist basic helix-loop-helix transcription factor 2	1.751665
PH_hs_0045784	**MT1A**	metallothionein 1A	1.659662
PH_hs_0018184	**MT1X**	metallothionein 1X	1.651276
PH_hs_0032121	ARL6	ADP-ribosylation factor-like 6	−4.519956
PH_hs_0036082	HIST1H3J	histone cluster 1, H3j	−3.96339
PH_hs_0042831	SPINK14	serine peptidase inhibitor, Kazal type 14 (putative)	−2.858793
PH_hs_0032938	C16orf47	chromosome 16 open reading frame 47	−2.674934
PH_hs_0002738	PGR	progesterone receptor	−2.653247
PH_hs_0019504	MZB1	marginal zone B and B1 cell-specific protein	−2.615926
PH_hs_0031538	RNASE11	ribonuclease, RNase A family, 11 (non-active)	−2.606224
PH_hs_0031319	SLA	Src-like-adaptor	−2.571705
PH_hs_0042528	OR51Q1	olfactory receptor, family 51, subfamily Q, member 1	−2.56117
PH_hs_0045727	FLT1	fms-related tyrosine kinase 1	−2.365311

MT genes were highlighted in bold.

MT expression can be induced by various types of factors, such as heavy metals (e.g. zinc, cadmium, nickel, copper, silver, and cobalt), glucocorticoids, alkylating agents, oxidizing agents, and inflammatory signals [38]. Our results showed for the first time that EZH2 inhibitors can induce MT expression. The induction of MT genes can be regulated by the metal-regulatory transcription factor MTF-1 through binding to the metal regulatory elements (MREs) on MT gene promoters [38]. Because the elevated zinc ion is required for the MRE-binding activity of MTF-1 [39], whether zinc ion is responsible for the GSK343-induced MT expression was investigated by using an zinc chelator, TPEN. As shown in Figure 3E, TPEN abolished GSK343-induced MT1/MT2A protein expression. In addition, TPEN rescued HepG2 cells from GSK343-induced cytotoxicity (Figure 3F). These results suggested that GSK343 may induced MT expression in a zinc-dependent manner, and the induction of MTs was associated with the anticancer activity of GSK343.

Recently, MT is thought to play a role in the pathogenesis of HCC [40]. Several MT1 subtypes, including MT1F, MT1G, MT1H, and MT1M, have been identified as tumor suppressor genes in a variety of cancers including HCC [41-46]. The expression of MT1G and MT1M is frequently silenced in HCC due to promoter hypermethylation [42, 43]. In contrast, 5-azacytidine, a DNA demethylating agent, can activate MTF-1 and induce MT expression [47, 48]. Because EZH2 can directly control DNA methylation through the recruitment of DNA methyltransferase (DNMT) to certain PRC2 target gene promoters [49], inhibition of EZH2 may activate MTF-1- dependent MT induction.

### Negative association of EZH2 and MT genes in cell lines and cancer tissues

**Figure 4:**
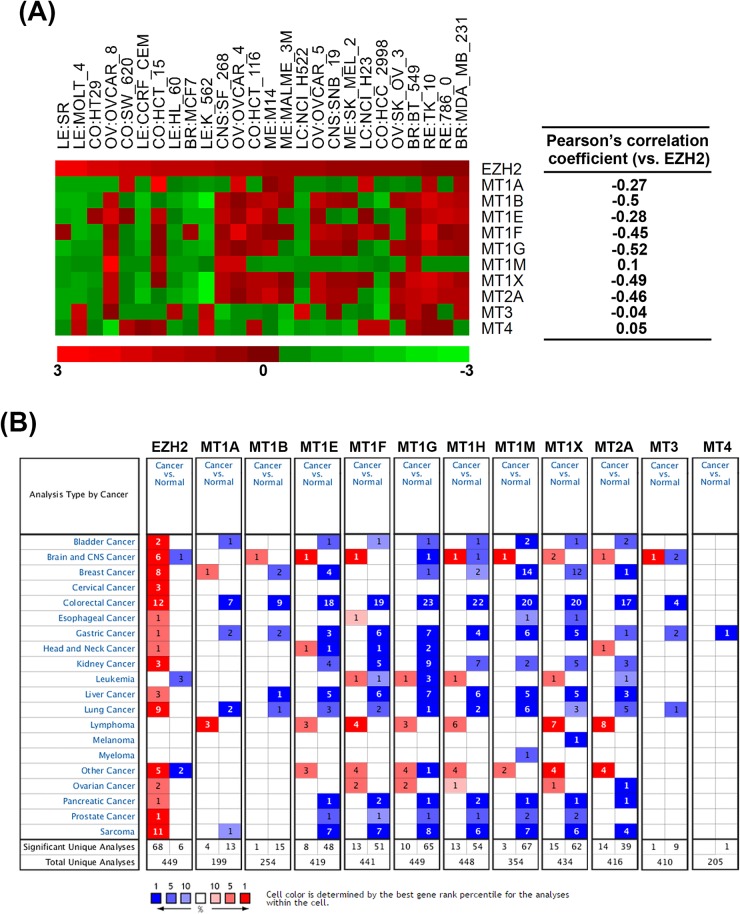
The correlation of EZH2 and MT genes in cell lines and cancer tissues **A.** Left part: Heat map shows the relative expression level of EZH2, MT1 genes, MT2A, MT3 and MT4 from 26 selected NCI-60 cell lines. Red square: increased expression; green square: decreased expression. Right part: Pearson's correlation between EZH2 and MT genes. Coefficient > 0: positive correlation. Coefficient < 0: negative correlation. Coefficient near 0 indicates no correlation between two genes. **B.** Summary view of EZH2 and MT gene expression profiles in human tumors using published human oncology microarray data (Oncomine, http://www.oncomine.org/). The number in each cell under “Cancer *vs*. Normal” corresponds to the amount of cancer types that contains a significantly different level of genes compared to normal corresponding tissue. Thresholds for significance are: fold expression > 2; *p*-value < 0.05 and ranking of gene in the analyses > top 10%. Red signifies the gene overexpression in the analyses; blue represents the gene underexpression. Intensity of color signifies the best rank of gene in those analyses.

To investigate the correlation of MTs and EZH2, the related expression levels of EZH2 and MT genes from 26 selected NCI-60 cell lines (average transcript intensity Z scores of EZH2 > 0.1) were retrieved from the CellMiner database (http://discover.nci.nih.gov/cellminer/) [50, 51]. Heat map and Pearson's correlation between EZH2 and MT genes showed the negative correlation between EZH2 and MT1 subtypes except for MT1M (Figure 4A). In addition, MT2A, but not MT3 and MT4, is also negatively correlated with EZH2 expression (Figure 4A), which was consistent with the results that GSK343 only induced mRNA expression of MT1 subtypes and MT2A (Figure 3C). To investigate the clinical relevance of EZH2 and MTs in cancers, we perform a pan-cancer analysis for the expressions of EZH2 and MT genes in normal and cancerous tissues by using a complete collection of human cancer microarray data (Oncomine Database, http://www.oncomine.org/) [26]. As shown in Figure 4B, EZH2 was frequently overexpressed in various cancer datasets (68 out of 449 analyses). Oppositely, MT1/MT2A, but not MT3/MT4, genes were frequently downregulated in cancer datasets, especially for MT1E (48 out of 419 analyses), MT1F (51 out of 441 analyses), MT1G (65 out of 449 analyses), MT1H (54 out of 448 analyses), MT1M (67 out of 354 analyses), MT1X (62 out of 434 analyses), and MT2A (39 out of 416 analyses). Therefore, these results suggest the negative association of EZH2 and MT1/MT2A in cancers including HCC. To further confirm the relationship between EZH2 and MTs in HCC, the expressions of MT genes were correlated with the level of EZH2 in three microarray datasets having both HCC and normal liver tissues [27-29]. Consistently, most MT1/MT2A genes were downregulated in two of these datasets, which was associated with higher expression of EZH2 (Table 3).

**Table 3:** The gene expression levels of EZH2 and MTs in HCC tumor tissues in three published microarray datasets

Gene Symbol	Fold Change (Chen X et al.)	Fold Change (Patil MA et al.)	Fold Change (Chen YL et al.)
EZH2	1.924	1.952	2.565
MT1A	ND	ND	ND
MT1B	−4.006	ND	ND
MT1E	−8.946	ND	−3.262
MT1F	−11.149	ND	−4.306
MT1G	ND	ND	−3.146
MT1H	−13.237	ND	ND
MT1M	ND	ND	−3.097
MT1X	−10.636	ND	−3.359
MT2A	ND	ND	−2.303
MT3	ND	ND	ND
MT4	ND	ND	−0.171

## CONCLUSION

Our *in silico* analysis shows that EZH2 is an attractive molecular target for treating HCC. Our *in vitro* study indicates that GSK343 is a potential anti-HCC agent. Microarray analysis finds that MT1 and MT2A genes are induced by GSK343, which is associated with its anticancer activity. In addition, negative association of EZH2 and MT1/MT2A expression is found in cancers including HCC. Therefore, we proposed that the reversion of these gene expressions profiles by GSK343 will provide therapeutic benefits. Taken together, these results demonstrate the therapeutic value of GSK343 for treating HCC through the induction of MT genes.

## MATERIALS AND METHODS

### Materials

MT1/MT2A and GAPDH antibodies were purchased form GeneTex (Hsinchu, Taiwan). Horseradish peroxidase-labeled goat anti-rabbit and anti-mouse secondary antibodies were purchased from Jackson ImmunoResearch (West Grove, PA, USA). DMEM medium, L-glutamine, sodium pyruvate, and Antibiotic- Antimycotic (penicillin G, streptomycin and amphotericin B) were purchased from Life Technologies (Gaithersburg, MD, USA). Fetal bovine serum (FBS) was purchased from GIBCO (Grand Island, NY, USA). GSK343 was purchased from Biovision (Mountain View, CA, USA). N,N,N',N'-tetrakis(2-pyridinylmethyl)-1,2-ethanediamine (TPEN) was purchased from Cayman Chemical (Ann Arbor, MI, USA). 3-(4,5-dimethylthiazol-2-yl)-2,5- diphenyl tetrazolium bromide (MTT) and dimethyl sulfoxide (DMSO) were purchased from Sigma (St. Louis, MO, USA). Protease and phosphatase inhibitor cocktails were purchased from Roche (Indianapolis, IN, USA). Other chemicals or reagents not specified were purchased from OneStar Biotechnology (New Taipei City, Taiwan).

### Cell culture

HepG2 cells were purchased from the Bioresources Collection and Research Center (BCRC), Food Industry Research and Development Institute (Hsinchu, Taiwan). Cells were cultured in DMEM medium supplemented with 10% FBS, 1 mM sodium pyruvate, 1% L-glutamine, 1% Antibiotic-Antimycotic Solution, and incubated at 37°C in a humidified incubator containing 5% CO2.

### Cell viability assay

Cell viability was measured with an MTT assay. Cells were plated in 96-well plates and treated with drugs. After 72 h of incubation, 0.5 mg/mL of MTT was added to each well for an additional 4 h. The blue MTT formazan precipitate was then dissolved in 200 μL of DMSO. The absorbance at 550 nm was measured on a multiwell plate reader.

### Western blot analysis

Cells were lysed in an ice-cold buffer containing 50 mM Tris-HCl (pH 7.5), 150 mM NaCl, 1 mM MgCl_2_, 2 mM EDTA, 1% NP-40, 10% glycerol, 1 mM DTT, 1x protease inhibitor cocktail and 1x phosphatase inhibitor cocktail at 4°C for 30 min. Cell lysates were separated on a sodium dodecylsulfate (SDS)-polyacrylamide gel, and then transferred electrophoretically onto the Hybond-C Extra nitrocellulose membrane (GE Healthcare, Piscataway, NJ, USA). The membrane was pre-hybridized in 20 mM Tris-HCl (pH 7.5), 150 mM NaCl, 0.05% Tween-20 (TBST buffer), and 5% skim milk for 1 h, and then transferred to a solution containing 1% bovine serum albumin (BSA)/TBST and a primary antibody and incubated overnight at 4°C. After washing with the TBST buffer, the membrane was submerged in 1% BSA/TBST containing a horseradish peroxidase-conjugated secondary antibody for 1 h. The membrane was washed with TBST buffer, and then developed with an enhanced chemiluminescence (ECL) system (Perkin-Elmer, Boston, MA, USA) and exposed to x-ray film (Roche, Indianapolis, IN, USA).

### Microarray analysis and real-time PCR

Total RNA was extracted from HepG2 cells that were treated with 10 μM GSK343 for 6 h. The mRNA profiles were analyzed using Human OneArray Plus (Phalanx Biotech, Hsinchu, Taiwan). Target genes were validated by real-time PCR. Total RNA was extracted by using TRIZOL reagent (Invitrogen, Carlsbad, CA, USA). The quantity of RNA samples was determined using NanoDrop ND-1000 (Thermo Scientific, Wilmington, DE, USA). RNA samples were reverse-transcribed for 120 min at 37°C with High Capacity cDNA Reverse Transcription Kit (Applied Biosystems, Foster City, CA, USA). Quantitative PCR was performed by the condition: 10 min at 95°C, and 40 cycles of 15 sec at 95°C, 1 min at 60°C using 2X Power SYBR Green PCR Master Mix (Applied Biosystems) and 200 nM of forward and reverse primers. Each assay was run on an Applied Biosystems 7300 Real-Time PCR system in triplicates and expression fold-changes were derived using the comparative CT method, GAPDH gene as endogenous control and untreated control sample as calibrator. The sequences of the primers used in real-time PCR were 5′-CCCATGAGCTGTGCCAAGT-3′ and 5′-TTCCAAGTTTGTGCAGGTCACT-3′ for MT1A, 5′-GCCCAGGGCTGCATCTG-3′ and 5′-TTCCAAGTTTGTGCAGGTCACT-3′ for MT1B, 5′-GGCTCCATTCTGCTTTCCAA-3′ and 5′-AGTGGCGCAAGAGCAGTTG-3′ for MT1E, 5′-AGCGGCCGGCTGTTG-3′ and 5′-AGAGACTGGACTTTCCAAGAGAGAAG-3′ for MT1F, 5′-CCTGTGCCGCTGGTGTCT-3′ and 5′-TGCAGCCTTGGGCACACT-3′ for MT1G, 5′-GGGCTGCATCTGCAAAGG-3′ and 5′-TTACGTGTCATTCTGTTTTCATCTGA-3′ for MT1H, 5′-CGCTCCATTTATCGCTTGAGA-3′ and 5′-TGCAGGCGCAGGAGACA-3′ for MT1M, 5′-GCTGCGTGTTTTCCTCTTGAT-3′ and 5′-GAGCAGCAGCTCTTCTTGCA-3′ for MT1X, 5′-GCCCAGGGCTGCATCTG-3′ and 5′-TTTGTGGAAGTCGCGTTCTTT-3′ for MT2A, 5′-CTGCCCCTGCCCTTCTG-3′ and 5′-ACACAGTCCTTGGCACACTTCTC-3′ for MT3, and 5′-GCAACTGTAAAACATGTCGGAAGA-3′ and 5′-AGCCTCCTTTGCAGATGCA-3′.

### Statistical analysis

Means and standard deviations of samples were calculated from the numerical data generated in this study. Data were analyzed using Student's *t* test. *p* values < 0.05 (*) were considered significant.

## SUPPLEMENTARY MATERIAL TABLE


